# Postural Control and Executive Functions in Individuals With Intellectual Disabilities: A Cross‐Sectional Study

**DOI:** 10.1002/brb3.71263

**Published:** 2026-02-24

**Authors:** Eva Fernández‐Baró, Jesús Seco‐Calvo, María Mercedes Reguera‐García, Ignacio Diez‐Vega

**Affiliations:** ^1^ Asprona Bierzo Ponferrada Spain; ^2^ ENSADE Research Group, Department of Nursing and Physiotherapy, Faculty of Health Sciences Universidad De León Ponferrada Spain; ^3^ Institute of Biomedicine (BIOMED) University of León León Spain; ^4^ Health and Quality of Life (HeQoL) Research Group, Department of Nursing and Physiotherapy, Faculty of Health Sciences Universidad De León Ponferrada Spain; ^5^ Exercise, Health and Applied Biomarkers Research Group Universidad Europea de Madrid Madrid Spain

**Keywords:** executive function, intellectual disability, mediolateral stability, postural balance

## Abstract

**Objective:**

The aim of the study was to compare the postural control of individuals with intellectual disabilities (ID) according to their level of executive function, considering different visual and postural conditions and center of pressure (COP) measures. Design: A cross‐sectional study.

**Setting:**

The participants were users of sheltered housing services and day centers.

**Participants:**

45 adults with ID.

**Interventions:**

Not Applicable.

**Outcome measures:**

The data collected included COP measures under various visual and postural conditions, as well as executive function measures in tests of cognitive flexibility, working memory, and inhibitory control. The data were analyzed using mixed analysis of variance (ANOVA).

**Results:**

Differences in postural control were observed according to the level of executive function and between the closed‐base, eyes‐open condition (CBEO) and the open‐base, eyes‐open (OBEO) and open‐base, eyes‐closed (OBEC) conditions (*p* < 0.01; η^2^_p_ > 0.14).

**Conclusion:**

Individuals with ID who exhibited lower executive function performance showed larger anteroposterior and mediolateral displacements, as well as increased COP area and velocity.

## Graphical abstract

This cross‐sectional study compared postural control in people with intellectual disabilities according to their level of executive functions, considering different visual and postural conditions. Lower executive function is associated with greater postural instability (increased anteroposterior and mediolateral displacements, area, and COP velocity).

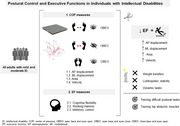

## 1 Introduction

Individuals with intellectual disability (ID) have difficulties achieving safe mobility and exhibit impairments in postural control (Enkelaar et al. 2012). The incidence of falls per year in adults with ID reaches 25%, with prevalence increasing with age (Hsieh et al. 2012). The causes of reduced postural control are multifactorial (Leyssens et al. 2022), and cognitive function impairments have been studied (Enkelaar et al. 2012; Horvat et al. 2013). In populations with mild and borderline ID, 19–23% of the variance in motor functions can be explained by cognition (Hallemans et al. 2019).

Maintaining quiet standing requires cognitive resources (Salihu et al. 2022). Athletes with ID do not alter their COP displacements in a dual‐task context compared to a single‐task condition, but their cognitive task performance decreases (Pineda et al. 2022). Comparing postural control in athletes with ID under different visual and postural conditions has demonstrated lower performance in single‐leg stance (greater COP area, length, and velocity), with 25% of participants completing the test with their eyes open and 15% with their eyes closed (Leyssens et al. 2022). The prioritization of the postural task may be a compensatory strategy for impaired sensorimotor processing (Doumas et al. 2008). Postural control limitations in individuals with Down syndrome could be related to insufficient cognitive resource allocation to process sensory information (Bieć et al. 2014).

The relationship between cognitive function and postural control involves neural resources from the prefrontal cortex, cerebellum, and basal ganglia, engaging processes such as planning, response inhibition, and working memory, collectively known as executive functions (Horvat et al. 2013; Stuhr et al. 2018). Postural control and executive functions share developmental trajectories between the ages of 5 and 10, continuing into adolescence (Hartman et al. 2010). Executive functions are cognitive processes involved in adaptive and goal‐directed behavior, essential for movement control (Stuhr et al. 2018). Individuals with ID exhibit executive dysfunctions from childhood (Ball et al. 2008; Costanzo et al. 2013), which become more pronounced with age (Grieco et al. 2015). Core executive functions include cognitive flexibility (the ability to modify behavior in changing situations) (Diamond 2013; Jurado and Rosselli 2007), working memory (the system for short‐term storage and manipulation of information) (Baddeley 1998), and inhibitory control (the ability to actively inhibit or delay a dominant response) (Diamond 2013; Spaniol and Danielsson 2022).

The relationship between executive functions and postural control is inconclusive (Stuhr et al. 2018). Correlation analyses reveal strong to moderate relationships between cognitive performance and COP measures in individuals with ID. The number of correct responses in the inhibition test is negatively correlated with COP velocity and variability in the anteroposterior (AP) direction (Azadian and Jabar Ali 2025). The review by Divandari et al. (2023) suggests that the executive function most closely associated with postural control is inhibitory control in healthy older adults. Other research points to cognitive flexibility (Stuhr et al. 2018) or working memory in multiple sclerosis (Perrochon et al. 2017).

Although cognitive deficits are evident in individuals with ID, it is unclear to what extent these deficits affect postural control. The available evidence in adults with ID is limited and shows inconsistent results, due to factors such as small sample sizes, methodological variability, and the lack of studies comparing postural stability according to the level of executive functions. Therefore, the present study aims to analyze differences in postural control based on the level of executive functions, considering different conditions (visual and postural) and assessment measures (anteroposterior and mediolateral displacements, COP area, and velocity), in order to determine the relationship between these two domains. It is expected that the results of this study will provide valuable information on how limitations in executive functions contribute to postural instability and guide specific interventions.

## 2 Method

### 2.1 Study Design and Sample

This is a cross‐sectional descriptive study with participants selected by convenience from an association of individuals with ID in Spain. The participants were users of sheltered housing services and day centers. The study was performed following the STROBE Statement.

The sample size calculation was performed using G*power 3.1.9.7. Unpublished results from a pilot study with six participants (*n* = 6) were used to estimate the effect size. Considering that an effect size (η^2^p) of 0.15 was obtained in the mixed ANOVA (2 groups and 3 measures of analysis), an alpha error probability (α) of 0.05, and a power (1‐β) of 0.8, a sample of 32 participants was deemed necessary.

The inclusion criteria were (a) being of legal age; (b) having a diagnosis of ID with intermittent or limited support needs; (c) autonomous standing ability; (d) ability to understand simple commands; and (e) signing of the informed consent form approved by the Ethics Committee of the University of León. The exclusion criteria were (a) using assistive devices for standing or walking, (b) current traumatic pathology, (c) conditions affecting postural control, and (d) significant hearing or expressive language impairments.

Participants were previously informed about the objectives and evaluation procedure. If they agreed to participate, in accordance with the Declaration of Helsinki (rev. 2013), they signed an informed consent form. Obtaining the signature of the informed consent was carried out following the provisions of Law 8/2021, of June 2, which establishes (art. 249 CC) that the exercise of capacity must be primarily direct, and in the event that support is required, it must be provided “taking into account the will, wishes, and preferences of the person who requires it.” In cases where necessary, the presence of a mediating judge was required, who explained the procedure to the subject according to their capacities, gave the final go‐ahead, and signed the consent. The institutional review board approved the study protocol and granted approval from the Ethics Committee of the University of León (ETICA‐ULE‐032‐2022).

### 2.2 Experimental Procedure

Data were collected across three separate sessions by a single researcher, with a maximum interval of one week between sessions, conducted between March and May 2023. Data coding and subsequent analysis were carried out by another researcher.

In the initial session, personal information and medical history were collected. The Montreal Cognitive Assessment (MOCA) (Nasreddine et al. 2005), used with permission obtained through the platform https://mocacognition.com/permission/ and the Supports Intensity Scale (SIS) (Verdugo Alonso et al. 2007) were administered with the participants' primary caregivers. The MOCA is an assessment of cognitive impairment, with a total score of 30 points, where scores below 25 indicate a potential cognitive disorder. The SIS assesses the intensity of support needs in daily activities. Classification categories are established: intermittent level: < 84 points, limited level: 85–99 points, extensive level: 100–115 points, generalized level: > 116 points (Verdugo Alonso et al. 2007). In the second session, descriptive variables and executive function tests were recorded. In the third session, COP measurements were collected.

### 2.3 Study Variables and Measurement Instruments

#### 2.3.1 Postural Control

The COP displacement was recorded in the anteroposterior (AP) (cm) and mediolateral (ML) (cm) directions, as well as the area covered by the trajectory with a 95% confidence interval (cm^2^) and velocity (cm/s) (Chen et al. 2021).

**FIGURE 1:**
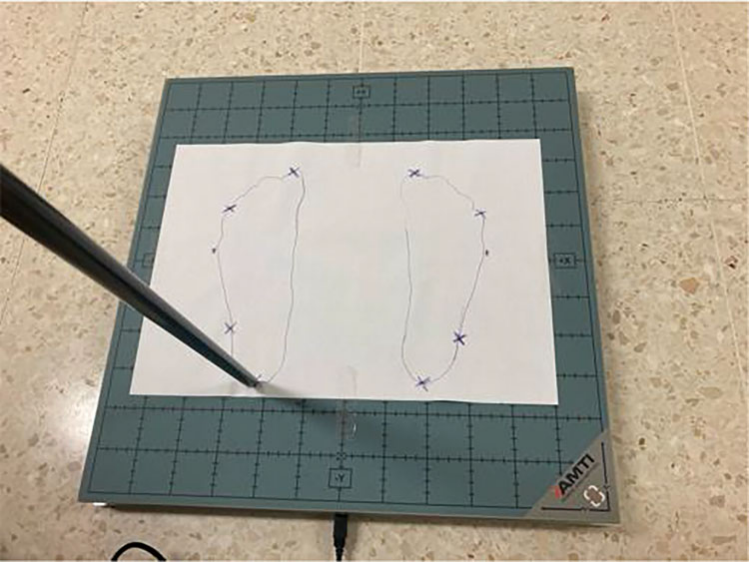
AccuSway Optimized platform, Advanced Mechanical Technology Inc. (AMTI), with foot contour template.

The force platform used was the AccuSway Optimized model, Advanced Mechanical Technology Inc. (AMTI), Watertown, MA, USA, with the “Balance Clinic” software. The sampling frequency was 100 Hz (Blomqvist et al. 2012; Pineda et al. 2020). (Figure 1).

The instructions were to remain in quiet standing, barefoot, as still as possible, with arms alongside the body and gaze fixed on a cross placed at eye level, 1.5 meters away. Three 30‐second trials were performed for each test, with a 1‐minute rest between trials (Pineda et al. 2020).

**FIGURE 2:**
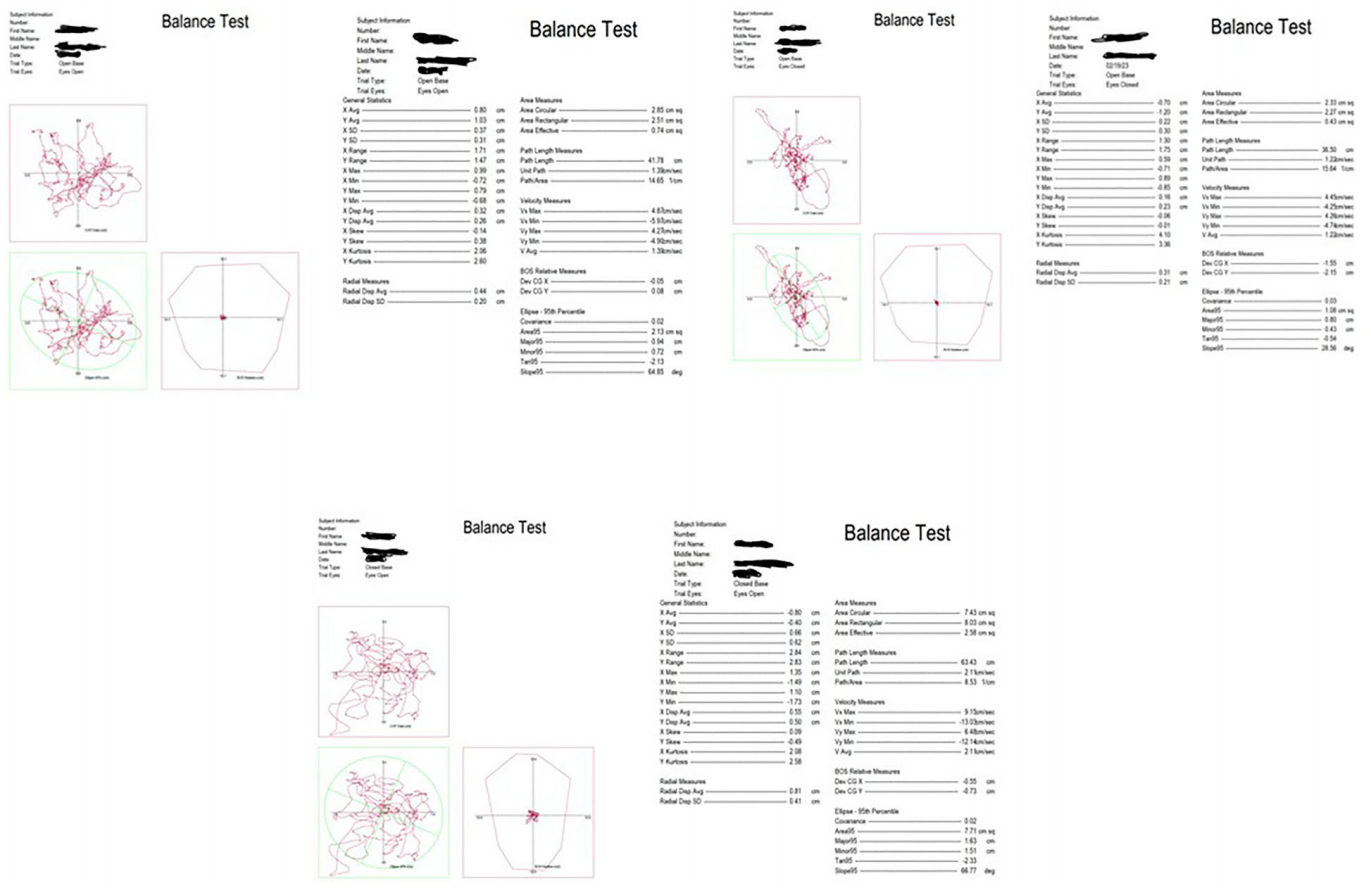
Report of tests from the AMTI platform with open base and eyes open (OBEO), open base and eyes closed (OBEC), and closed base and eyes open (CBEO).

Visual (eyes open or closed) and postural conditions (standing with parallel feet at an intermalleolar distance of 10 cm or standing with feet together) were modified, following this order: (a) open base, eyes open (OBEO), (b) open base, eyes closed (OBEC), (c) closed base, eyes open (CBEO). (Figure 2).

An acceptable reliability of COP measures in individuals with ID has been described following a similar protocol (Pineda et al. 2020).

#### 2.3.2 Executive Functions

The results of three executive function tests were recorded.

- Cognitive flexibility (CF): Semantic verbal fluency task (number of unique animals named in one minute) (Ball et al. 2008; Diamond 2013). Scores were awarded as follows: 0 points for “0 animals,” 1 point for “1–4 animals,” 2 points for “5–9 animals,” 3 points for “10–14 animals,” 4 points for “15–19 animals,” and 5 points for “more than 20 animals” (Ball et al. 2008; Diamond 2013; Willner et al. 2010). This test is considered to have low dependency on literacy skills (Rowe et al. 2006) and has demonstrated good reliability (ICC = 0.72) in Down syndrome (Smeyne et al. 2022).
- Working memory (WM): Backward digit span test (Baddeley 1998). This test involves memorizing and repeating a sequence of numbers in reverse order. Each difficulty level has two attempts with different numbers. Two points are awarded if both attempts are correct, 1 point if one attempt is correct, and 0 points if both attempts fail. The maximum score was 14 points (Diamond 2013; Wechsler 2001). This test has shown validity in detecting cognitive impairment in Down syndrome and sufficient reliability in adults with neurocognitive disorders (ICC = 0.59) (Hartley et al. 2017).
- Inhibitory control (IC): Cats and dogs task (Ball et al. 2008). The examiner presents a sequence of 16 images of dogs and cats in a predetermined order. The participant responds ‘dog’ when the image is a cat and “cat” when it is a dog. One point was awarded for each correct response (Willner et al. 2010). This test is considered appropriate (Kristensen et al. 2022) and has demonstrated sufficient reliability (ICC = 0.46) in Down syndrome (Schworer et al. 2023).

### 2.4 Statistical Analysis

The distributions of the variables were examined, and the fulfillment of parametric assumptions was verified.

Tertiles were used to divide participants into two groups (low and high level) based on their executive function test results, eliminating the middle‐level group from the analysis to maximize differences between the created groups.

To analyze differences in postural control based on executive function levels (low and high), evaluation conditions (OBEO, OBEC, CBEO), COP measures (AP displacement, ML displacement, area, and velocity), and the interaction between these factors, a 2 × 3 × 4 mixed analysis of variance (ANOVA) was used. For each factor, a post‐hoc analysis with Bonferroni correction was performed. The significance level was set at p<0.05. Effect size was analyzed using partial eta squared (η^2^_p_), considering small (η^2^_p_ = 0.01), medium (η^2^_p_ = 0.06), and large (η^2^_p_ = 0.14) effect sizes (Lakens 2013).

Statistical analysis was performed using SPSS v. 29.

## 3 Results

### 3.1 Descriptive Characteristics of the Participants

**FIGURE 3:**
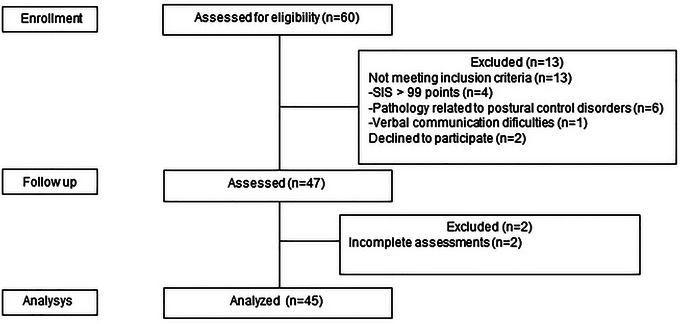
Flowchart diagram.

A total of 45 individuals participated in this study (28 men and 17 women), with a level of mild (*n* = 31) and moderate (*n* = 14) ID. (Figure 3).

The participants were adults (37.07 ± 10.27 years), overweight (28.67 ± 6.63 kg/m^2^), with cognitive impairment (MOCA 17.07 ± 7.62), and an intermittent or limited level of support needs (SIS 83.53 ± 7.84) (Table 1).

**TABLE 1:** Descriptive and clinical characteristics of participants.

Age, years (mean ± SD)	37.07 ± 10.27
Sex, *n* (%)	
Male	28 (62.22)
Female	17 (37.7)
Level of ID, *n* (%)	
Mild	31 (68.88)
Moderate	14 (31.11)
Severe	0 (0)
Medical history, *n* (%)	
Neurological pathology	6 (13.33)
Pharmaceuticals with CNS effects	27 (60.00)
Traumatological antecedents	7 (15.55)
Musculoskeletal pathology	6 (13.33)
Visual impairment	10 (22.22)
Auditory impairment	4 (8.88)
Weight, kg (mean ± SD)	77.40 ± 17.96
Height, cm (mean ± SD)	164.58 ± 9.35
BMI, kg/m^2^ (mean ± SD)	28.67 ± 6.64
MOCA, points (mean ± SD)	17.07 ± 7.63
SIS, points (mean ± SD)	83.53 ± 7.84

**Abbreviations**: BMI, body mass index; CNS, central nervous system; MOCA, Montreal cognitive assessment; SIS, Support Intensity Scale.

### 3.2 Executive Function Test Results

Table 2 shows the results of the executive function tests for the low‐ and high‐level groups, the number of participants in each group, and the significance level (*p* < 0.001). The feasibility of the verbal fluency, working memory, and inhibitory control tests was 100%, 82.22%, and 93.33%, respectively.

**TABLE 2:** Results of the executive function tests according to level.

	All		Low‐level		High‐level		
	n	x̅ ± SD	n	x̅ ± SD	n	x̅ ± SD	p
CF	45	3.02 ± 1.16	14	1.64 ± 0.50	15	4.33 ± 0.49	<0.001
WM	45	3.67 ± 2.60	18	1.11 ± 1.02	18	6.28 ± 1.49	<0.001
IC	45	13.71 ± 4.48	15	9.47 ± 5.81	25	16.00 ± 0.00	<0.001

**Abbreviations**: CF, cognitive flexibility; IC, inhibitory control; WM, working memory.

### 3.3 Postural Control Results According to Executive Function Level, Evaluation Condition, COP Measures, and Interactions

The analysis of variance revealed significant differences in postural control based on the level of executive functions, evaluation condition, COP measure, and interactions between these factors. Participants with a lower level of executive function showed greater AP and ML displacement, a larger area, and higher COP velocity compared to the high executive function group.

Table 3 summarizes the postural control results based on the level of cognitive flexibility (CF). Significant differences were observed according to CF level (*F* (1) = 8.68; *p* = 0.007; η^2^_p_ = 0.25) and evaluation condition (*F* (2) = 39.27; *p* < 0.001; η^2^_p_ = 0.60), as well as in the interaction between measure × CF (*F* (3) = 5.32; *p* = 0.002; η^2^_p_ = 0.17). There were no significant interactions for condition × CF (*F* (2) = 0.9; *p* = 0.414; η^2^_p_ = 0.03), nor for condition × measure × CF (*F* (6) = 1.48; *p* = 0.19; η^2^_p_ = 0.05).

**TABLE 3:** Descriptive statistics and analysis of variance according to level of cognitive flexibility.

				CF		Condition		Condition × CF		Measure × CF		Condition × Measure × CF	
		Low‐level^L^	High‐level^H^	*p*	η^2^_p_	*p*	η^2^_p_	p	η^2^_p_	*p*	η^2^_p_	*p*	η^2^_p_
AP^a^	OBEO^1^	2.6 ± 0.62	2.37 ± 0.72	**0.007**	**0.25**	**< 0.001 1–3; 2‐3**	**0.60**	0.41 **1: L‐H** **2: L‐H** **3: L‐H** **L:1‐3;2‐3** **H:1‐3;2‐3**	0.03	**0.002** **a: L‐H** **b: L‐H** **c: L‐H**	**0.17**	0.39 **L:1‐2; 1–3**	0.03
	OBEC^2^	3.23 ± 1.24	2.40 ± 0.62									**0.03**	**0.17**
	CBEO^3^	3.34 ± 0.99	2.83 ± 0.66									0.11	0.09
ML^b^	OBEO^1^	1.94 ± 0.74	1.30 ± 0.37									**<0.001 L: 1–3; 2–3** **H: 1–3; 2–3**	**0.25**
	OBEC^2^	1.73 ± 0.63	1.30 ± 0.43									**0.04**	**0.15**
	CBEO^3^	3.65 ± 1.13	2.93 ± 0.57									**0.04**	**0.16**
Area^c^	OBEO^1^	3.53 ± 1.49	2.23 ± 1.19									**0.02 L: 1–3; 2–3** **H: 1–3; 2–3**	**0.20**
	OBEC^2^	4.13 ± 3.37	2.27 ± 1.27									0.06	0.13
	CBEO^3^	9.57 ± 6.11	6.19 ± 1.99									**0.05**	**0.14**
V^d^	OBEO^1^	1.51 ± 0.29	1.30 ± 0.28									0.07 **L: 1–3; 2–3** **H: 1–3; 2–3**	0.12
	OBEC^2^	1.70 ± 0.47	1.42 ± 0.40									0.10	0.10
	CBEO^3^	2.17 ± 0.58	1.80 ± 0.49									0.08	0.11

CF, cognitive flexibility; AP, anteroposterior displacement (^a^); ML, mediolateral displacement (^b^); Area (^c^); V, velocity (^d^); OBEO, open base‐ open eyes (^1^); OBEC, open base‐ close eyes (^2^); CBEO, close base‐ open eyes (^3^); ^L^: low level CF; ^H^: high level CF; *p*, *p*‐value (in bold significant differences); η^2^_p_: partial eta squared.

The post‐hoc analysis identified better postural control in the OBEO condition compared to CBEO (*p* < 0.001) and in OBEC compared to CBEO (*p* < 0.001). No differences were observed between OBEO and OBEC (*p* = 1).

Individuals with a high level of CF exhibited better postural control than those with low CF (*p* = 0.007) in AP displacement (p = 0.041), ML displacement (*p* = 0.005), and area (*p* = 0.014), but not in velocity (*p* = 0.053).

For AP displacement, the greatest difference between groups was observed in the OBEC condition, with the low CF group showing greater AP displacement between OBEO‐OBEC and OBEO‐CBEO. Differences in ML displacement were observed across all evaluation conditions. COP area and velocity were greater in the low CF group, particularly in more complex conditions.

Table 4 summarizes the postural control results based on the level of working memory (WM). Significant differences were observed according to WM level (*F* (1) = 16.98; *p* < 0.001; η^2^_p_ = 0.35), and evaluation condition (*F* (1.70) = 63.35; *p* < 0.001; η^2^_p_ = 0.66), as well as in the interaction between measure × WM (*F* (3) = 9.72; *p* < 0.001; η^2^_p_ = 0.23). There were no significant interactions for condition × WM (*F* (2) = 1.95; *p* = 0.15; η^2^_p_ = 0.06), nor for condition × measure × WM (*F* (6) = 1.60; *p* = 0.15; η^2^_p_ = 0.05).

**TABLE 4:** Descriptive statistics and analysis of variance according to level of working memory.

				WM		Condition		Condition × WM		Measure × WM		Condition × Measure × WM	
		Low‐level^L^	High‐level^H^	*p*	η^2^_p_	*p*	η^2^_p_	*p*	η^2^_p_	*p*	η^2^_p_	*p*	η^2^_p_
AP^a^	OBEO^1^	2.52 ± 0.64	2.19 ± 0.71	**< 0.001**	**0.35**	**<0.001 1–3; 2‐3**	**0.66**	0.15 **1:L‐H** **2:L‐H** **3:L‐H** **L:1‐3;2‐3** **H:1‐3;2‐3**	0.06	**< 0.001 a: L‐H** **b: L‐H** **c: L‐H** **d: L‐H**	**0.23**	0.17 **L:1‐3** **H:1‐3**	0.06
	OBEC^2^	3.03 ± 1.22	2.24 ± 0.50									**0.02**	**0.16**
	CBEO^3^	3.40 ± 0.85	2.66 ± 0.68									**< 0.001**	**0.20**
ML^b^	OBEO^1^	1.80 ± 0.76	1.14 ± 0.26									**< 0.001** **L: 1–3; 2–3** **H: 1–3; 2–3**	**0.27**
	OBEC^2^	1.80 ± 0.61	1.09 ± 0.29									**< 0.001**	**0.38**
	CBEO^3^	3.65 ± 0.86	2.78 ± 0.67									**< 0.001**	**0.25**
AREA^c^	OBEO^1^	3.33 ± 1.58	1.77 ± 0.97									**<0.001 L: 1–3; 2–3** **H: 1–3; 2–3**	**0.28**
	OBEC^2^	4.15 ± 3.15	1.65 ± 0.65									**< 0.001**	**0.25**
	CBEO^3^	9.11 ± 5.41	5.49 ± 2.12									**< 0.001**	**0.20**
V^d^	OBEO^1^	1.46 ± 0.26	1.27 ± 0.29									0.06 **L:1‐2; 1‐3;2‐3** **H: 1–3; 2–3**	0.11
	OBEC^2^	1.67 ± 0.45	1.36 ± 0.31									**0.03**	**0.14**
	CBEO^3^	2.29 ± 0.51	1.75 ± 0.42									**< 0.001**	**0.27**

WM: working memory; AP: anteroposterior displacement (^a^); ML: mediolateral displacement (^b^); Area (^c^); V: velocity (^d^); OBEO: open base‐ open eyes (^1^); OBEC: open base‐ close eyes (^2^); CBEO: close base‐ open eyes (^3^); ^L^: low level WM; ^H^: high level WM; p: p‐value (in bold significant differences); η^2^_p_: partial eta squared.

The post‐hoc analysis identified better postural control in the OBEO condition compared to CBEO (*p* < 0.001) and in OBEC compared to CBEO (*p* < 0.001). However, no differences were observed between OBEO and OBEC (*p* = 0.65).

Individuals with a high level of WM exhibited better postural control than those with low WM (*p* < 0.001) in AP displacement (*p* = 0.007), ML displacement (*p* < 0.001), area (*p* < 0.001), and velocity (*p* = 0.004).

For AP displacement, the greatest difference between groups was observed in the CBEO condition, with a larger increase in AP displacement between OBEO and CBEO conditions. Differences in ML displacement and area were observed across all evaluation conditions. Differences in velocity were observed in the OBEC and CBEO conditions, with the low WM group showing higher results in all conditions.

Table 5 summarizes the postural control results based on the level of inhibitory control (IC).

**TABLE 5:** Descriptive statistics and analysis of variance according to level of inhibitory control.

				IC		Condition		Condition× IC		Measure× IC		Condition× Measure × IC	
		Low‐level^L^	High‐level^H^	*p*	η^2^_p_	*p*	η^2^_p_	*p*	η^2^_p_	*p*	η^2^_p_	*p*	η^2^_p_
AP^a^	OBEO^1^	2.49 ± 0.54	2.25 ± 0.69	**<0.001**	**0.29**	**<0.001 1–3;2‐3**	**0.70**	**<0.001** **1:L‐H** **2:L‐H** **3:L‐H** **L:1‐3; 2‐3** **H:1‐3;2‐3**	**0.15**	**<0.001** **a: L‐H** **b: L‐H** **c: L‐H**	**0.28**	0.29 **L:1‐3** **H:1‐3**	0.03
	OBEC^2^	2.91 ± 1.19	2.37 ± 0.64									0.08	0.09
	CBEO^3^	3.44 ± 0.93	2.75 ± 0.72									**0.02**	**0.15**
ML^b^	OBEO^1^	1.87 ± 0.67	1.26 ± 0.52									**<0.001 L: 1–3; 2–3** **H: 1–3; 2–3**	**0.21**
	OBEC^2^	1.80 ± 0.59	1.20 ± 0.45									**<0.001**	**0.26**
	CBEO^3^	3.99 ± 0.94	2.75 ± 0.59									**<0.001**	**0.41**
AREA^c^	OBEO^1^	3.26 ± 1.18	2.09 ± 1.44									**0.02** **L: 1–3; 2–3** **H: 1–3; 2–3**	**0.15**
	OBEC^2^	4.04 ± 3.35	2.04 ± 1.26									**0.01**	**0.17**
	CBEO^3^	10.30 ± 5.70	5.62 ± 2.17									**<0.001**	**0.27**
V^d^	OBEO^1^	1.37 ± 0.22	1.34 ± 0.33									0.82 **L:1‐3; 2–3** **H:1‐2; 1–3; 2–3**	<0.01
	OBEC^2^	1.49 ± 0.31	1.47 ± 0.38									0.87	<0.01
	CBEO^3^	2.17 ± 0.52	1.86 ± 0.50									0.08	0.08

IC: inhibitory control; AP: anteroposterior displacement (^a^); ML: mediolateral displacement (^b^); Area (^c^); V: velocity (^d^); OBEO: open base‐ open eyes (^1^); OBEC: open base‐ close eyes (^2^); CBEO: close base‐ open eyes (^3^); ^L^: low level IC; ^H^: high level IC; p: p‐value (in bold significant differences); η^2^_p_: partial eta squared.

Significant differences were observed according to IC level (*F* (1) = 14.78; *p* < 0.001; η^2^_p_ = 0.29) and evaluation condition (*F* (1.69) = 83.34; *p* < 0.01; η^2^_p_ = 0.70), as well as in the interactions between condition × IC (*F* (2) = 6.15; *p* < 0.001; η^2^_p_ = 0.15), measure × IC (*F* (3) = 13.71; *p* < 0.001; η^2^_p_ = 0.28), and condition × measure × IC (*F* (6) = 6.11; *p* < 0.001; η^2^_p_ = 0.15).

The post‐hoc analysis identified better postural control in the OBEO condition compared to CBEO (*p* < 0.001) and in OBEC compared to CBEO (*p* < 0.001), with no differences found between OBEO and OBEC (*p* = 0.68).

Individuals with a high level of IC exhibited better postural control than those with low IC (*p* < 0.001) in AP displacement (*p* = 0.027), ML displacement (*p* < 0.001), and area (*p* < 0.001), but not in velocity (*p* = 0.314).

For AP displacement, the greatest difference between groups occurred in the CBEO condition, with a larger AP displacement between OBEO and CBEO conditions. Differences in ML displacement and area were observed across all evaluation conditions. In velocity, the low IC group showed worse results between OBEO‐CBEO and OBEC‐CBEO conditions, while the high IC group showed superior velocity across all conditions.

## 4 Discussion

The objective of this study was to analyze differences in postural control in adults with ID, examining the level of executive functions, visual and postural evaluation conditions, COP measures, and their interactions.

The COP area and velocity in the OBEO and OBEC conditions were similar to those reported in sedentary young adults with mild ID and higher than those observed in athletes with ID (Jouira et al. 2021; Pineda et al. 2020). However, velocity values were lower than those reported in individuals with moderate ID (Jouira et al. 2024). Although the sample included participants with moderate ID, 68.9% were individuals with mild ID, exhibiting higher cognitive and adaptive functioning. In the CBEO condition, COP area and velocity values were also comparable to those of adults with ID (Leyssens et al. 2022) and higher than those reported in adolescents with ID (Blomqvist et al. 2012). Age‐related declines contribute to reduced postural stability (Goble and Baweja 2018; Julienne et al. 2024), and the presence of premature aging in individuals with ID may further exacerbate this effect (Enkelaar et al. 2012).

Our results show the association between executive functions and postural control. Poor performance in executive functions results in significantly greater AP and ML displacement, COP area, and velocity, and therefore, more deficient postural control (Chen et al. 2021). In individuals with cognitive impairment, the decline in executive functioning and motor performance is associated with atrophy in regions such as the prefrontal cortex (Fastame et al. 2022). In individuals with ID, the prefrontal cortex and connecting structures may show alterations from birth (Muñoz‐Ruata, 2013), which are exacerbated by aging (Teipel and Hampel, 2006). Therefore, postural control difficulties in individuals with ID are associated with deficits in executive functions (Pineda et al. 2022) due to alterations in brain structures.

The CBEO condition identifies more differences between levels of executive function. The reduction in the base of support increases COP displacements and velocity in the ML direction (Henry et al. 2001), requiring more postural adjustments to avoid reaching postural limits (Chiari et al. 2002). However, the OBEC condition does not detect differences between groups. In individuals with ID, the visual system does not appear to be the dominant system for postural control (Blomqvist et al. 2012). Some studies suggest that the OBEC condition has lower reliability than other conditions (Bahureksa et al. 2017).

ML displacement was the measure that detected significant differences between groups for all executive functions and evaluation conditions. ML postural control is managed through the hip strategy, which involves modulation of hip abductor activity and the proximal‐to‐distal activation of other muscle groups. Therefore, alterations in this mechanism could limit postural control in this direction (Morasso 2022). Due to limited exposure to tasks that challenge ML postural control (Morasso 2022), the integration and automation of this skill do not occur (Aslan et al. 2021), making it more dependent on cognitive resources.

COP velocity indicates the activity required to maintain postural stability per unit of time, so lower values imply active control of COP dynamics (Palmieri et al. 2002). Our participants with lower cognitive performance did not significantly increase their velocity in response to greater COP displacements, resulting in slower and less effective postural adjustments (Rodrigo Antonio et al. 2011).

Our results indicate deficits in ML postural control and an inability to generate rapid postural adjustments in individuals with ID, particularly those with lower executive functioning. These deficits lead to difficulties in weight transfers, a reduction in lumbopelvic stability, and ineffective responses in dynamic tasks (Addison et al. 2017). Therefore, training the hip abductor muscles or postural tasks with a reduced base of support could be effective strategies. Due to the bidirectional relationship between executive functions and motor skills (Bao et al. 2024), practicing cognitively demanding postural tasks may positively influence the improvement of executive functions.

This study presents some methodological limitations related to the inability to use nonlinear variables that reflect the automation and cognitive involvement in postural tasks (Pineda et al. 2022). In addition, the absence of a control group and convenience sampling should be considered as factors that limit the generalization of the results.

The results achieved in this study directly address the initial objective by demonstrating that postural control in adults with ID varies according to their level of executive functioning. Future research could focus on studying the effectiveness of ML postural control training programs in standing to enhance the efficacy of the hip strategy, improve sensory processing, and influence cognition in individuals with ID. These findings indicate the importance of integrating cognitive and motor training programs for individuals with ID.

## 5 Conclusions

Postural control in adults with ID varies according to the level of executive functions. Individuals with lower executive functioning exhibit greater AP and ML COP displacement, larger COP area, and higher COP velocity, particularly in the CBEO.

The findings suggest that interventions focused on improving ML postural control, strengthening the hip abductor muscles, and performing cognitively challenging postural tasks could enhance both postural stability and executive functions in individuals with ID.

## Author Contributions

E. F. B., J. S. C., M. M. R. G., and I. D. V. contributed to the conceptualization and design of the study. E. F. B. and M. M. R. G. developed the data collection instruments and collected the data. I. D. V., J. S. C., and E. F. B. performed the initial analyses. E. F. B., M. M. R. G., and I. D. V. drafted the manuscript. All authors critically reviewed the manuscript to ensure its intellectual content, approved the final version for submission, and agree to be accountable for all aspects of the work.

## Declaration of Generative AI and AI‐assisted Technologies in the Writing Process

During the preparation of this work, the authors used ChatGPT 4° payment in order to improve the translation of the manuscript. After using this tool/service, the authors reviewed and edited the content as needed and take full responsibility for the content of the publication.

## Funding

This research received funding from the IX Research Grant of the Professional College of Physiotherapists of Castilla y León (Spain).

## Ethics Statement

The study was conducted according to the guidelines of the Declaration of Helsinki and approved by the Ethics Committee of the University of León (protocol code: ETICA‐ULE‐032‐2022).

## Consent

Informed consent was obtained from all subjects involved in the study.

## Conflicts of Interest

The authors declare no conflict of interest.

## Data Availability

The data presented in this study are available on request from the corresponding author.
